# High-density genetic linkage mapping in Sitka spruce advances the integration of genomic resources in conifers

**DOI:** 10.1093/g3journal/jkae020

**Published:** 2024-02-15

**Authors:** Hayley Tumas, Joana J Ilska, Sebastien Gérardi, Jerome Laroche, Stuart A’Hara, Brian Boyle, Mateja Janes, Paul McLean, Gustavo Lopez, Steve J Lee, Joan Cottrell, Gregor Gorjanc, Jean Bousquet, John A Woolliams, John J MacKay

**Affiliations:** Department of Biology, University of Oxford, Oxford OX1 3RB, UK; The Roslin Institute, Royal (Dick) School of Veterinary Science, University of Edinburgh, Midlothian EH25 9RG, UK; Canada Research Chair in Forest Genomics, Forest Research Centre, Université Laval, Québec, QC GIV 0A6, Canada; Institute for Systems and Integrative Biology, Université Laval, Québec, QC GIV 0A6, Canada; Institute for Systems and Integrative Biology, Université Laval, Québec, QC GIV 0A6, Canada; Forest Research, Northern Research Station, Midlothian EH25 9SY, UK; Institute for Systems and Integrative Biology, Université Laval, Québec, QC GIV 0A6, Canada; The Roslin Institute, Royal (Dick) School of Veterinary Science, University of Edinburgh, Midlothian EH25 9RG, UK; Forest Research, Northern Research Station, Midlothian EH25 9SY, UK; Forest Research, Northern Research Station, Midlothian EH25 9SY, UK; Forest Research, Northern Research Station, Midlothian EH25 9SY, UK; Forest Research, Northern Research Station, Midlothian EH25 9SY, UK; The Roslin Institute, Royal (Dick) School of Veterinary Science, University of Edinburgh, Midlothian EH25 9RG, UK; Canada Research Chair in Forest Genomics, Forest Research Centre, Université Laval, Québec, QC GIV 0A6, Canada; Institute for Systems and Integrative Biology, Université Laval, Québec, QC GIV 0A6, Canada; The Roslin Institute, Royal (Dick) School of Veterinary Science, University of Edinburgh, Midlothian EH25 9RG, UK; Department of Biology, University of Oxford, Oxford OX1 3RB, UK

**Keywords:** exome capture, genetic map integration, comparative genomics, Pinaceae, SNP discovery, Plant Genetics and Genomics

## Abstract

In species with large and complex genomes such as conifers, dense linkage maps are a useful resource for supporting genome assembly and laying the genomic groundwork at the structural, populational, and functional levels. However, most of the 600+ extant conifer species still lack extensive genotyping resources, which hampers the development of high-density linkage maps. In this study, we developed a linkage map relying on 21,570 single nucleotide polymorphism (SNP) markers in Sitka spruce (*Picea sitchensis* [Bong.] Carr.), a long-lived conifer from western North America that is widely planted for productive forestry in the British Isles. We used a single-step mapping approach to efficiently combine RAD-seq and genotyping array SNP data for 528 individuals from 2 full-sib families. As expected for spruce taxa, the saturated map contained 12 linkages groups with a total length of 2,142 cM. The positioning of 5,414 unique gene coding sequences allowed us to compare our map with that of other Pinaceae species, which provided evidence for high levels of synteny and gene order conservation in this family. We then developed an integrated map for *P. sitchensis* and *Picea glauca* based on 27,052 markers and 11,609 gene sequences. Altogether, these 2 linkage maps, the accompanying catalog of 286,159 SNPs and the genotyping chip developed, herein, open new perspectives for a variety of fundamental and more applied research objectives, such as for the improvement of spruce genome assemblies, or for marker-assisted sustainable management of genetic resources in Sitka spruce and related species.

## Introduction

Recombination frequency analysis was developed over a century ago to order genetic markers (Sturtevant 1913), leading to the development of genetic linkage maps and ultimately the linking of phenotypic traits to chromosomal regions. Genetic linkage mapping (e.g. Gyapay *et al*. 1994), along with high-throughput DNA sequencing, was instrumental in producing the first human genome sequence assembly (Lander *et al*. 2001). In plants, linkage maps allowed for positioning gene coding regions and anchoring sequence scaffolds obtained through whole-genome sequencing in a variety of species including poplar (Tuskan *et al*. 2006), potato (Xu *et al*. 2011), eucalypt (Myburg *et al*. 2014), ryegrass (Velmurugan *et al*. 2016), soybean (Song *et al*. 2016), or spruces (Gagalova *et al*. 2022). Linkage maps are useful for laying the genomic groundwork in species with genomes that are difficult to assemble due to size or complexity, such as barley (5.1 Gb) and wheat (16 Gb) (Mascher *et al*. 2013; Chapman et al. 2015), which both have large hexaploid genomes and abundant repetitive sequences. For this reason, the development of the first saturated linkage maps in conifers (e.g. Devey *et al*. 1994; Pelgas *et al*. 2006), which have very large genomes (18–34 Gb) and extensive repetitive regions (MacKay *et al*. 2012; De La Torre *et al*. 2019), predates by 2 decades the report of first genome assemblies (Birol *et al*. 2013; Nystedt *et al*. 2013; Zimin *et al*. 2014; Warren *et al*. 2015). Despite the rapid development of sequencing technologies, genetic linkage maps remain an essential genomic resource for species with such large genomes and highly fragmented genome assemblies (De La Torre *et al*. 2019). The importance of a wide number of conifer species in breeding programs and productive forestry across the globe (Mullin *et al*. 2011) has encouraged the development of genetic linkage maps and other genomic resources to support fundamental research and diverse applications (Bousquet *et al*. 2021).

One of the main findings emerging from comparative genome mapping studies in conifers has been the detection of high levels of intergeneric macro-synteny and macro-collinearity among Pinaceae taxa (Pelgas *et al*. 2006; Ritland *et al*. 2011; Pavy *et al*. 2012; Westbrook *et al*. 2015). The low incidence of large chromosomal rearrangements, despite the ancient divergence within the group, has enabled the development of consensus maps across species. For example, the high structural conservation in *Pinus taeda* L. and *Pinus elliottii* Engelm. enabled the development of a consensus genetic map with 3,856 markers (Westbrook *et al*. 2015) and similarly across *Picea glauca* (Moench) Voss and *Picea mariana* (Mill.) B.S.P. (Pavy *et al*. 2008, 2012). Likewise, highly conserved gene coding sequences among Pinaceae taxa have allowed to transfer efficiently exome capture sequencing probes across species; for instance, probes originally developed in *P. glauca* (Stival Sena *et al*. 2014 ) were successfully used for large-scale single nucleotide polymorphism (SNP) discovery in gene coding regions of *P. mariana* (Pavy *et al*. 2016) and *Picea abies* (L.) H. Karst (Azaiez *et al*. 2018).

However, most of the 600+ extant conifer species still lack linkage maps or have maps with a low marker density, limiting their usefulness in molecular breeding applications or other genomic analyses. Nonetheless, the recent advent of high-throughput genotyping techniques has allowed to use DNA markers covering thousands of genetic loci and to develop high-density linkage maps in several conifer and plant species. As for most forest trees, conifers have high levels of genetic diversity and heterozygosity (Hamrick and Godt 1990), which has facilitated the large-scale discovery of SNPs by expression tag sequencing (Dantec *et al*. 2004; Pavy *et al*. 2006), targeted resequencing by using exome capture (Neves *et al*. 2014; Pavy *et al*. 2016, Azaiez *et al*. 2018), and genotyping by sequencing (e.g. Gamal El-Dien *et al*. 2015). As a result, extensive genomic resources have been developed but only for a few commercially relevant Pinaceae taxa, such as *P. taeda* L. (Neves *et al*. 2014), *Pinus pinaster* Aiton (de Miguel *et al*. 2015; Plomion *et al*. 2016), *Pinus flexilis* (E. James) Rydberg (Liu *et al*. 2019), *P. glauca* (Pavy *et al*. 2013, 2017), *P. mariana* (Pavy *et al*. 2016), and *P. abies* (Bernhardsson *et al*. 2019). Several genotyping methods have been used in conifers, from custom chips (Pavy *et al*. 2008, 2013, 2016; Moriguchi *et al*. 2012; Plomion *et al*. 2016) to targeted sequencing (Neves *et al*. 2014; Bernhardsson *et al*. 2019) and reduced representation whole-genome sequencing (e.g. restriction site associated DNA sequencing (Gamal El-Dien *et al*. 2015)).

The reported high levels of genome synteny and collinearity among the Pinaceae provide an opportunity to accelerate the development of genomic resources in ecologically and economically relevant species. Sitka spruce (*Picea sitchensis* [Bong.] Carr.) still lacks a large-scale genotyping resource or a high-density linkage map but has a large database of mRNA sequences (Ralph *et al*. 2008) and a draft genome assembly (Gagalova *et al*. 2022). *P. sitchensis* is a long-lived conifer found mostly in the coastal areas of western North America, and that is widely planted for forestry in the British Isles (Lee *et al*. 2013). Linkage mapping and genomic selection are of great interest to improve our understanding of the genetic basis of quantitative traits appropriate for tree breeding (Lee *et al*. 2013; Fuentes-Utrilla *et al*. 2017) and genetic diversity management to maintain or increase resilience to damaging pests in *P. sitchensi*s in the context of exotic forestry and climate change (Tumas *et al*. 2021). Here, we aimed to develop genomic markers that could be used in conjunction with a comparative genomic approach to produce a genetic linkage map. Our specific objectives were as follows: (1) use probes developed in *P. glauca* (Stival Sena *et al*. 2014) to perform exome capture and SNP discovery in Sitka spruce; (2) develop a large-scale SNP array for genotyping natural and mapping Sitka populations; (3) develop high-density linkage maps from full-sib families by using data from the SNP array and previous restriction site associated DNA sequencing (RAD-seq) data (Ilska *et al*. 2023); (4) compare the resulting *P. sitchensis* linkage map to maps from those available for other conifers; and (5) develop an integrated *Picea* genetic map with *P. glauca*.

## Material and methods

### Study population, sampling, and DNA extraction

**Fig. 1.**
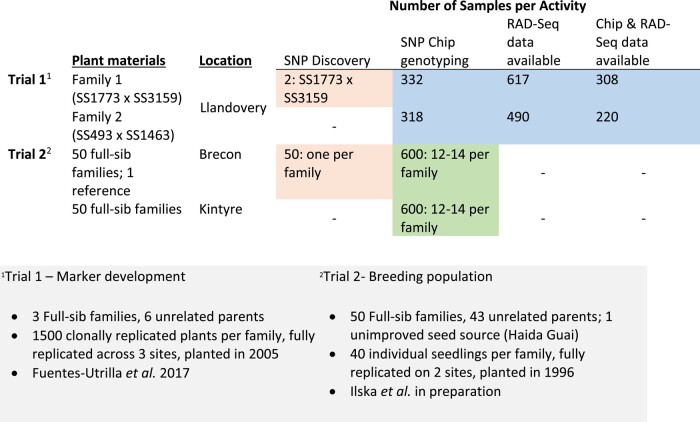
Details of the full-sib genetic trials (in gray) and the samples taken from these that were used in SNP discovery (column 3) for the SNP Chip and SNP Chip validation (column 4, rows 3–4), the subset of samples used for SNP Chip and RAD-seq genotyping to develop the linkage map (column 4-6, rows 1–2).

**Fig. 2.**
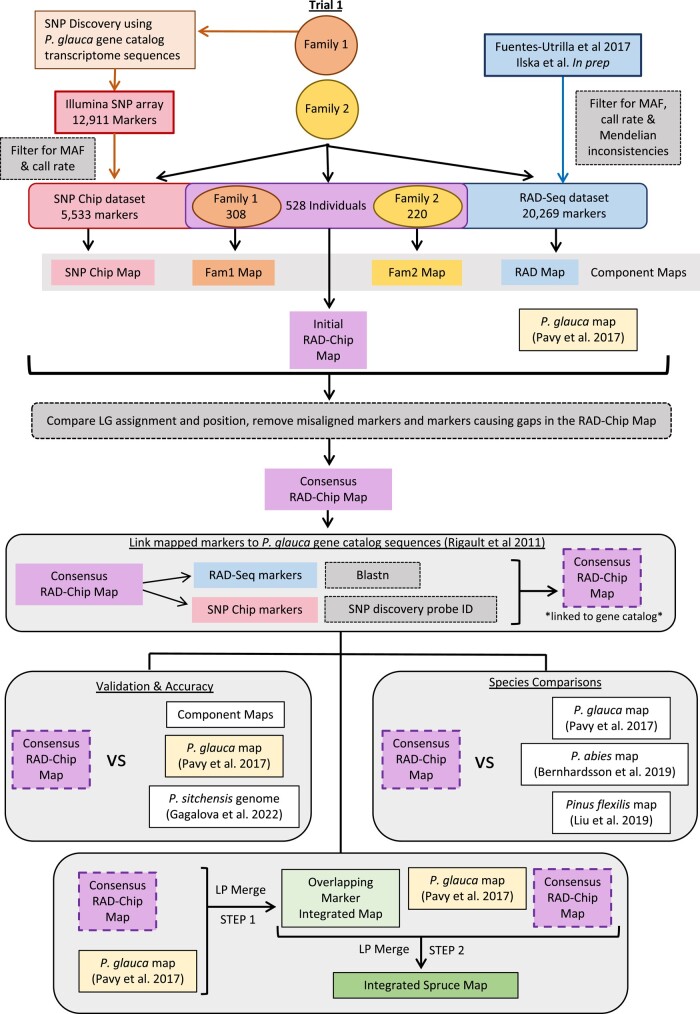
Schematic of the *P. sitchensis* map development, validation, species comparison, and map integration steps. Several *P. sitchensis* component maps were initially developed using each family and marker type; however, the final RAD-Chip map was developed as a single step combining both families and both the RAD-seq and the Infinium Chip data (Top). Markers in the RAD-Chip map were linked to *P. glauca* gene catalog sequences (dashed line) to facilitate comparative genomic analysis approaches (middle), which were then used to assess the accuracy of the map, conduct to study synteny across species, and then develop an integrated spruce map (bottom).

All plant materials in this study were from 2 distinct *P. sitchensis* full-sib genetic field trials (trial 1 and trial 2) established in the United Kingdom. Information on the trials is presented in Fig. 1, along with details on samples used for (1) SNP discovery (orange), (2) SNP Chip validation (green), or (3) linkage map development (blue), and which samples in trial 1 had additional RAD-seq genotyping data (Fuentes-Utrilla *et al*. 2017) used in linkage map development (Fig. 2). Briefly, trial 1, consisting of 3 full-sib families replicated across 3 sites, was used for SNP discovery and linkage map development while trial 2 comprised 50 full-sib families across 2 sites and was used for SNP discovery and validation in this study and to develop genomic prediction in a separate study (Ilska *et al.* 2023). Samples from 2 full-sib families in trial 1 (family 1: SS1773 × SS3159; family 2: SS493 × SS1463) were used in linkage map development and were all collected from a single site in Llandovery, United Kingdom (Fuentes-Utrilla *et al*. 2017), for genotyping using either the SNP Chip, RAD-seq, or both methods (Fig. 1).

The sampling for SNP discovery and SNP Chip genotyping was carried out in July to August 2017, whereas the RAD-seq sampling was completed as described previously in Fuentes-Utrilla *et al*. (2017). All samples were comprised of foliage from healthy annual growth collected by removing 1–3 shoot tips of approximately 5–10 cm in length from healthy branches, subsequently placing them intact in sealed, labeled plastic bags and storing them in a cool box for less than 48 h. After transporting them to the laboratory, the needles were removed from the rachis and stored at −20°C until used for DNA isolation.

DNA was isolated for SNP discovery from parents of the linkage mapping families in trial 1 and 1 randomly selected individual for each of the 50 full-sib families in trial 2 by Forest Research. Needles (100 mg) were finely chopped, placed in 2-ml eppendorf tubes with two 3-mm stainless steel ball bearings, and ground to a fine powder in a Retsch mixer mill (Retsch, Haan, Germany). DNA was isolated from powder using a Qiagen DNAeasy Plant mini kit (QIAGEN, Hilden, Germany) with the following modifications. Lysis buffer AP1 volume was increased from 400 to 600 µl, and incubation time was increased from 10 to 20 min. The neutralization buffer (P3) volume was increased from 130 to 200 µl, and a constant volume of 800 µL of AW1 wash buffer was added to each sample. During the elution step, the eluted product was reapplied to the column, incubated for 5 min, and then spun down to elute the final product. DNA concentration was measured using a Qubit fluorometer (original model, Thermo Fisher Scientific, MA, USA). DNA for the SNP Chip was isolated from 50 mg of needles by the Austrian Institute of Technology (AIT, Vienna, Austria). DNA isolation for RAD-seq genotyping was described in Fuentes-Utrilla *et al*. (2017).

### Exome capture sequencing

Samples from 2 parents of 1 family in trial 1 (SS1773 × SS3159) and a pool of samples comprised of 1 individual of each of the 50 families in trial 2 (Fig. 1) were used as libraries for SNP discovery to develop the SNP Chip (Fig. 2). The trial 2 pool was assembled by merging untagged extracted DNAs from each individual in equimolar concentration. One large insert (avg. 650 bp) NEBNext Ultra II library (New England Biolabs, Ipswich, MA, USA) was generated for each of the 2 trial 1 parents (SS1773 and × SS3159) and for the pool of trial 2 samples, following the manufacturer's instructions.

Oligonucleotide probes used herein to capture *P. sitchensis* gene homologs were originally designed from *P. glauca* gene catalog sequences (Rigault *et al*. 2011) and were previously used successfully under an exome capture framework on *P. glauca* (Stival Sena *et al*. 2014), *P. mariana* (Pavy *et al*. 2016), and *P. abies* (Azaiez *et al*. 2018). Multiple probes (0.5 M) ranging from 50 to 105 nucleotides in length were designed for each of the 23,684 transcripts of the white spruce gene catalog, with each base being covered by 2 probes on average. This approach affords a reduced representation analysis of *Picea* spp. genomes, which are around 20 Gb in size (Gagalova *et al*. 2022). Two micrograms of libraries (100 ng from each parent and 900 ng from the breeding population) were used in a liquid-phase capture (SeqCap EZ developer, IRN 6089042357, OID35086, Roche NimbleGen). The captured material was amplified and sequenced on an Illumina HiSeq 4000 PE100 at the Centre d’Expertises et de Services Génome Québec (Montréal, QC, Canada). Illumina HiSeq 4000 sequencing generated two ∼100-bp paired-end sequences per captured insert, which yielded over 403 M raw sequences for the 3 libraries (Supplementary Table 1).

### Read library processing, reference-guided alignment, and SNP detection

For the reads obtained for each library, Illumina adapter sequences were removed using the software Cutadapt 2.7 (Martin 2011), and sequencing quality was checked before and after adapter removal with the software FastQC v0.11.8 (Andrews 2010). After this step, 100, 64, and ∼237 M sequences were obtained for the 2 trial 1 libraries (SS1773 and SS3159) and trial 2 library, respectively (Supplementary Table 1). Reads were then mapped to the most complete version of the white spruce (*P. glauca*) catalog of expressed genes (Laoué *et al*. 2021 ) initially described (Rigault *et al*. 2011), which contains 27,720 gene cluster sequences including the 23,684 sequences that were used to design the probes. *P. sitchensis* and *P. glauca* are closely related taxa that can hybridize (Hamilton *et al*. 2013); therefore, this strategy allowed to maximize gene representation and facilitate subsequent SNP selection for the design of a genotyping array (see *Genotyping assay* section below for further details). Each library was aligned to the reference gene catalog using the BWA-MEM algorithm (Li and Durbin 2010) and was converted to BAM format with SAMtools (Li 2011). Around 25% of the sequences mapped to the gene catalog, representing a total of over 100 M mapped sequences (Table 1), with the other 75% of sequences being off-target with low read depth. Next, variant calling was performed with the software Platypus v0.8.1 (Rimmer *et al*. 2014). A minimum read depth of ≥25 was used for variant calling, and all remaining criteria were Platypus default parameters (for details, see Supplementary Data 1). Variant calling with Platypus resulted in the identification of 286,159 SNPs distributed across 23,480 GCAT gene clusters.

**Table 1.** Library sequencing and read mapping*^a^* summary statistics (Nb = numbers of).

Library	Nb. raw reads	Nb. reads post quality control	Supplementary*^b^*	Nb. mapped reads (%)
Trial 1 parent SS1773	100,001,026	99,811,140	161,959	24,576,905 (25)
Trial 1 parent SS3159	64,519,124	64,092,588	103,976	15,883,403 (25)
Trial 2 families	238,330,524	237,123,494	415,307	62,800,361 (26)
Total	402,850,674	401,027,222	681,242	103,260,669 (26)

^*a*^The reads were mapped to the latest version of the reference gene catalog for *P. glauca* (Laoué *et al*. 2021) that was used to design the sequence capture probes as described (Stival Sena *et al*. 2018).

^*b*^For reads that aligned to 2 sequences in the reference chimerically, 1 segment was designated as primary, and the remainder as supplementary.

### SNP Chip assay and genotyping

The SNPs discovered here were used along with 1,554 SNPs already identified in *P. sitchensis* from a previous genotyping study using *P. glauca* Infinium genotyping arrays (Pavy *et al*. 2013) to develop a new Infinium iSelect array (Illumina, San Diego, CA, USA) for genomic analyses. All newly discovered SNPs retained for building the array met the following general criteria: (1) were strictly biallelic SNPs; (2) included only 1 SNP per gene and were type II SNPs (1 bead per SNP) whenever possible, in order to maximize the number of genes represented on the array; (3) carried no SNP or indel within 50 bp in their 5′ or 3′ flanking regions (Illumina probe design requirement); and (4) had Illumina functionality scores ≥ 0.4. More specifically, SNPs observed in at least 1 mapping parent library (SS1773 or SS3159) were retained when they met the following criteria: depth ≥ 25; minor allele frequency (MAF) ≥ 0.25 in trial 1 parent libraries, and MAF ≥ 0.05 in the trial 2 library. SNPs observed in the trial 2 library only were also selected when their MAF exceeded 0.15, and their depth exceeded 50 reads. For this last subset of SNPs, when more than 1 SNP was available for a given gene, the SNP with MAF around 0.25 was retained so as to filter out possible paralogs expected to yield MAFs close to 0.5.

Following chip manufacture by Illumina, genotypes were obtained both for the mapping families from trial 1 (analyzed in this report) and for the full-sib of trial 2 (analyzed in a separate study in preparation) at the Centre d’expertise et de services Génome Québec (Montréal, QC, Canada, group of Daniel Vincent). Genotype calling was performed using the GenomeStudio v2.0.5 software (Illumina), and genotype clusters were visually examined and manually curated when necessary to reject monomorphic and failed polymorphisms. Excel files output from GenomeStudio were formatted for PLINK v1.90b4 (Purcell *et al*. 2007) using R v4.0.2 (R Core Team 2022). Data for the trial 1 individuals were filtered separately in PLINK to retain only those SNPs with a minimum call rate of 80% and a MAF greater than 0.2 and to exclude individuals with a call rate below 85%, and then data were reformatted into variant calling files (VCFs). The data for the trial 2 individuals were also filtered in PLINK—mind (maximum missing data per sample) 0.05 and MAF greater than 0.05.

### Combining datasets

Data from the Infinium iSelect SNP array (SNP Chip dataset) were combined with a dataset from a previous study (Fuentes-Utrilla *et al*. 2017; Ilska *et al*. 2023) that used RAD-seq to genotype a similar set of samples from the same full-sib families in trial 1 (RAD-seq dataset) (Fig. 1). Following filtering for individual (60%) and SNP (80%) call rate, MAF (0.15), and Mendelian inconsistencies, the RAD-seq dataset contains 15,452 and 17,915 genotyped loci across 617 and 490 offspring and parents for families 1 and 2, respectively. Samples across the 2 families were combined in the RAD-seq dataset, and a single SNP per locus was chosen based on call rate for a total of 27,967 SNPs across 1,111 individuals. The SNP Chip and RAD-seq datasets were joined by overlap in sampled individuals with 308 and 220 individuals, including parents, overlapping in families 1 and 2, respectively for a total of 528 individuals in the combined dataset (Fig. 2). These overlapping individuals were extracted from VCFs containing each complete SNP Chip and RAD-seq dataset using VCFtools v0.1.16 (Danecek *et al*. 2011), and then resulting VCFs were merged using “concat” in BCFtools v1.8 (Danecek *et al*. 2021) to combine datasets for mapping.

### Constructing linkage maps

Linkage maps were constructed using Lep-MAP3 v0.2 (Rastas 2017) with Java v8.45.14. Lep-MAP3 allows family data to be combined and used simultaneously for construction of linkage maps. In total, 5 maps were constructed, 1 consensus map that is the main resource of this study and 4 component maps developed from different subsets of the data that were used for method validation (Fig. 2). The consensus map (RAD-Chip Map) used both families 1 and 2 and the combined SNP Chip and RAD-seq marker dataset. The 4 component maps were constructed separately, 2 using both families but each of the marker datasets separately (SNP Chip and RAD maps) and 2 using the combined marker dataset but only samples from each family separately (Fam1 and Fam2 maps, corresponding to family 1 and family 2 in Fig. 1). Data for each of these 5 maps were input into Lep-MAP3 using “ParentCall2” allowing the removal of noninformative markers (removeNonInforamtive = 1) and then filtered using “Filtering2” using the default data tolerance for segregation distortion of 0.01. Markers were assigned to linkage groups (chromosomes) using “SeparateChromosomes2,” testing a range of minima for the logarithm of the odds score between groups of markers (lodLimit) between 15 and 95 and with a minimum of 100 markers set as the requirement to form a group. When developing the 2 family component maps (Fam1 and Fam2), Fam2 had a linkage group length distribution in centimorgans more comparable to that found in *P. glauca* (Pavy *et al*. 2017) while Fam1 had a much longer first linkage group exceeding 247 cM. For this reason, data for family 2 were used to inform marker grouping for the other 3 maps that combined families (RAD-Chip, SNP Chip, and RAD maps) using the “families” function within “SeparateChromosomes2.” Markers that were not assigned to a linkage group were then added to these generated linkage groups using “JoinSingles2All” by testing a range of lodLimits from 2 to 50 and using a lodDifference of 10. The best lodLimit was selected for each step by determining which value assigned the most markers to 12 linkage groups, the known number of chromosomes in *P. sitchensis* (Supplementary Table 1). Markers were ordered on linkage groups and relative position in centimorgans was determined using “OrderMarkers2” with the Kosambi mapping function (useKosambi = 1) and averaging marker position over sex (sexAveraged = 1). This ordering step was iterated 5 times for each chromosome, and the order with the highest likelihood was selected as the final map for each dataset or family.

The consensus RAD-Chip map was further developed by removing problematic markers, which were identified by examining gaps at the end of linkage groups and by checking for inconsistencies in linkage group assignment and order of markers or genes in the RAD-Chip map compared to the 4 other component maps (SNP Chip, RAD, Fam1, and Fam2) as well as the *P. glauca* map (Pavy *et al*. 2017). Gaps were identified visually by plotting the RAD-Chip map in ggplot2 (Wickham 2016) in R v4.03 (R Core Team 2022). Differences in linkage group assignments were determined by merging resulting maps and aggregating by linkage group in R v4.03. Marker order was compared between maps using linear models with the “lm” function in R v4.03, based on the idea that, when plotted against one another, marker positions within a linkage group should have a linear relationship when maps have similar marker order. Using only markers that grouped the same across the 2 maps, the position of the marker in the component or *P. glauca* map was regressed against the position in the RAD-Chip map for each linkage group. Cook's distance (Cook 1977) was used to identify any markers that had a substantially different position in the 2 maps, using a threshold of 4/*n*, where *n* is the number of markers in the comparison. Lep-MAP3 was rerun on the RAD-Chip dataset as described above using only those markers that mapped previously, excluding markers that caused gaps at the end of linkage groups, were assigned to different linkage groups in 2 or more map comparisons, or markers that surpassed the Cook's distance threshold in any comparison.

### Map validation and accuracy

Marker linkage group assignment and order in the consensus RAD-Chip map were validated against the 4 component maps (SNP Chip, RAD, Fam1, and Fam2) by calculating the proportion of markers assigned to the same linkage group and the correlation in marker order using Kendall's *τ* in R v4.03. The accuracy of the RAD-Chip map linkage group assignment and marker ordering was verified using the *P. glauca* gene catalog GCAT3.3 (Rigault *et al*. 2011) and the *P. sitchensis* genome sequence (Gagalova *et al*. 2022; GenBank assembly no. GCA_010110895.2). Map assignment and ordering were considered accurate if markers located on the same *P. glauca* gene or *P. sitchensis* genome scaffold were assigned to the same linkage group and located at the same position or within a window of 10 cM to one another. All SNP Chip SNPs have a corresponding *P. glauca* gene from using the *P. glauca* transcriptome sequences to inform exome capture in the SNP discovery. RAD-seq SNPs were matched to the *P. glauca* catalog using Blastn (Altschul *et al*. 1990; Camacho *et al*. 2008), calling reciprocal best hits with a minimum of 95% identity and a maximum *E*-value of 1 × 10^−11^. Sequences of *P. glauca* catalog genes with matches in the RAD-Chip map were used in Blastn to find matches in the *P. sitchensis* genome using reciprocal best hits with a minimum of 95% identity and a maximum *E*-value of 1 × 10^−100^. Linkage group and position were compared among any markers that were located on the same sequence or scaffold.

### Comparisons to other species

Synteny across other species within the *Pinaceae* family was examined using a consensus map from Norway spruce (*P. abies* (L.) Karst.) (Bernhardsson *et al*. 2019) and maps of *P. glauca* (Pavy *et al*. 2017) and limber pine (*P. flexilis* James) (Liu *et al*. 2019) (Fig. 2). The set of sequences from the *P. glauca* gene catalog (Rigault *et al*. 2011) matching to either SNP Chip markers or RAD-seq markers on the RAD-Chip map, determined either during the SNP discovery for the SNP Chip markers or with Blastn as described above for the RAD-seq markers, were used as the basis of comparison to the other species. Mapped markers in the *P. glauca* map all sit within a sequence in the *P. glauca* gene catalog (Rigault *et al*. 2011; Pavy *et al*. 2013; Pavy *et al*. 2017), allowing for direct comparison. For comparison to *P. abies* and *P. flexilis*, Blastn was used to find orthologous genes between *P. sitchensis* and the mapped genes in each species. Markers mapped in *P. abies* were identified using sequence capture based on the *P. abies* genome assembly v1.0 (Nystedt *et al*. 2013) available on PlantGenIE (https://plantgenie.org/), and markers mapped in *P. flexilis* were identified using sequence capture based on a *P. flexilis* transcriptome provided by Liu *et al*. (2019). Mapped sequences were extracted from these files for synteny analysis. Orthologous marker pairs were identified as reciprocal best hits with a maximum *E*-value of 1 × 10^−100^ and a minimum of 95 and 90% identity when comparing to *P. abies* genome scaffolds and *P. flexilis* transcriptome sequences, respectively. In the particular case when orthologous *P. abies* SNPs were located on the same genome scaffold, but assigned to different linkage groups according to the *P. abies* map, both SNPs were included in the synteny analyses and accounted for statistically. Synteny was evaluated both visually and statistically by calculating the proportion of orthologous genes that were assigned to the same linkage group and estimating the correlation in the marker order using Kendall's *τ*.

### Constructing an integrated Spruce map

Using the *P. glauca* gene catalog (Rigault *et al*. 2011) annotation associated with the *P. sitchensis* SNP Chip dataset and the markers on the *P. glauca* map along with the additional matches in the mapped RAD-seq markers as described above, *P. sitchensis* and *P. glauca* (Pavy *et al*. 2017) maps were integrated with the aim of developing a spruce map that included more genes than either of the species maps alone (Fig. 2). To simplify integration of gene placement across species and prevent inconsistencies among markers, a single marker per *P. glauca* gene catalog sequences was used. The *P. glauca* map only includes 1 marker per catalog gene, so, first, a single marker per gene catalog sequence was selected from the markers with a match to the catalog on the RAD-Chip map, preferentially selecting SNP Chip markers. Second, markers causing major discrepancies between the 2 maps were removed, i.e. markers that were not assigned to the same linkage group or markers that had surpassed the threshold for order misalignment using Cook's distance as described above. Filtered maps were then combined using “LPMerge” (Endelman and Plomion 2014) in R v4.03 in 2 steps.

In the first step, only markers found on both maps were used to create an integrated map, giving the maps weights equivalent to sample size and testing a maximum interval size between bins from 1 to 10 across each linkage group. The second step generated the final integrated species map by combining the filtered individual species maps with all markers with the resulting merged map from the first step, giving the filtered RAD-Chip and *P. glauca* maps and the merged map weights of 1, 2, and 3, respectively, to reflect sample size and confidence. In both steps, the best consensus map was selected across the interval size bins both by comparing the lowest root-mean-squared error for each linkage group and by comparing the integrated species map linkage group length to the average length of the RAD-Chip and *P. glauca* map linkage groups. In the integrated species map from step 2 containing all possible markers, large gaps were created at the ends of linkage groups. The markers creating these gaps were manually removed in the final integrated species map. Synteny with the component species maps and this final integrated species map was assessed by calculating the percentage of markers assigned to the same linkage group, the correlation in marker order using Kendall's *τ*, and visually through graphs in R.

## Results

### SNP Chip genotyping

The final SNP Chip array contained 12,911 markers across 12,893 unique sequences from the *P. glauca* gene catalog (see *Materials and methods* for identification and selection of SNPs). Of those markers, 1,554 were previously shown to be polymorphic in *P. sitchensis* according to an array designed for *P. glauca* (Pavy *et al*. 2013) and 4,604 had been previously mapped in *P. glauca* (Pavy *et al*. 2017). Following filtering for call rate and MAF (see *Materials and methods* for details), 5,533 markers were successfully called as informative across the 2 linkage mapping families, among which 2,572 markers were from the previously mapped in *P. glauca*. Similarly, 6,946 markers were successfully called as informative in a total of 1,262 individuals from trial 2 trees (Table 2).

**Table 2.** Summary of SNP Chip genotyping results across trials and SNP discovery populations.

SNP discovery population	Nb on Chip	Trial 1—informative	Trial 1—uninformative	Trial 2—informative	Trial 2—uninformative
Recycled	1,554	1,407 (95%)	147 (5%)	1,466 (94%)	88 (6%)
Mapping parents	5,303	1,828 (34%)	3,475 (66%)	2,375 (45%)	2,928 (55%)
50 F-S families	6,054	2,298 (38%)	3,756 (62%)	3,105 (51%)	2,949 (49%)
ALL	12,911	5,533 (43%)	7,378 (57%)	6,946 (54%)	5,965 (46%)

Informative SNPs were polymorphic after filtering for call rate and MAF (see *Materials and methods*); uninformative SNPs gave a low call rate, were monomorphic or polymorphic with a low MAF (Nb = numbers of).

### Linkage maps and map integrity

In the SNP Chip dataset, 3 and 8 offspring were removed from families 1 and 2, respectively, due to low call rate and 5,533 markers across the 2 families passed filtering for call rate and MAF in PLINK. Following further filtering in Lep-MAP3 for segregation distortion and uninformative markers, 615 samples (including of parents) and 5,194 markers in total were retained to develop the SNP Chip component map. Additional filtering in Lep-MAP3 reduced the RAD-seq dataset from 27,967 to 19,529 markers across 1,111 samples in both families for use in the RAD component map. The combined SNP Chip and RAD-seq datasets contained 25,802 markers, 5,533 from the SNP Chip dataset and 20,269 from the RAD-seq dataset, across the 528 individuals genotyped with both methods. Following additional filtering in Lep-MAP3, 24,702 markers were used to construct the RAD-Chip consensus map, 5,194 from the SNP Chip dataset and 19,508 from the RAD-seq dataset. The component family maps used 14,499 and 15,955 markers across 308 and 220 samples for Fam1 and Fam2, respectively, following filtering in Lep-MAP3.

As expected, all maps placed markers across 12 linkage groups. The component SNP Chip, RAD, Fam1, and Fam2 maps placed 5,064, 15,041, 12,685, and 14,831 markers, respectively, with an average total map length of 2,412.3 cM that ranged from 2,148.2 for the RAD map to 2,927.6 for the SNP Chip map. The initial RAD-Chip consensus map placed 22,505 markers with a total map length of 2,367.3 cM. A total of 934 markers were excluded from these 22,505 markers for causing gaps or consistent differences in assignment or order across comparisons to the component maps and the *P. glauca* map. The finalized RAD-Chip map that excluded these problematic markers mapped 21,570 markers for a total map length of 2,141.6 cM and an average distance of 0.1 cM between markers.

In comparison to the 4 component maps, 99.90–99.99% of markers were assigned to the same linkage group in the finalized RAD-Chip map with the least agreement occurring with the Fam1 map (Supplementary Fig. 1). Concordance in marker order ranged from 0.95–0.99 with the lowest correlation occurring with the Chip (Supplementary Fig. 1). When linking mapped markers to the *P. glauca* catalog, 4,590 genes were mapped from the SNP Chip dataset and 1,094 were mapped from the RAD-seq dataset, which resulted in a total of 5,414 unique genes positioned on the final map (270 overlapping genes between both datasets). Of these genes, 326 were linked to 2 or more markers for a total of 670 markers located on genes carrying at least 1 other marker. Across these 670 markers, 92% were assigned to the same linkage group as the co-occurring SNP with an average distance of 3 cM (0–157.2 cM) between co-occurring markers. When linking mapped markers to the *P. sitchensis* genome using *P. glauca* catalog sequences, 2,161 unique scaffolds were represented on the final map with 32 scaffolds matching more than 1 marker arising from the co-occurrence of 70 markers. Only 2 scaffolds had markers that were not assigned to the same linkage group with 94% of co-occurring markers assigned to the same linkage group and an average distance of 0.8 cM (0–2.7 cM) between co-occurring markers.

### Synteny across species

**Fig. 3.**
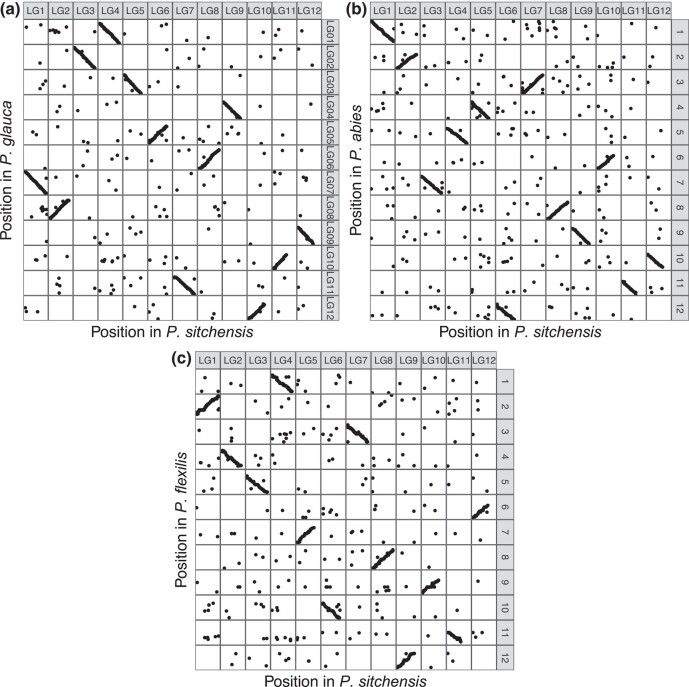
Comparison of marker assignment and order on chromosomes (linkage groups) between *P. sitchensis* (*x*-axis) and linkage maps in 3 other species from previous studies (*y*-axis): a) *P. glauca* (Pavy *et al*. 2017), b) *P. abies* (Bernhardsson *et al*. 2019), and c) *P. flexilis* (Liu *et al*. 2019). Note that the linkage group (LG) labels on the *y*-axis are taken from the originally published map in each species.

Synteny between the *P. sitchensis* consensus RAD-Chip map and *P. glauca* map (Pavy *et al*. 2017) was based on 2,581 catalog genes. These genes corresponded to 2,778 marker pairs on both maps with 94% of them being assigned to the same linkage group and with an average concordance in marker order of 0.98 (0.96–0.98) across linkage groups (Fig. 3b). A total of 3,234 marker pairs were used to compare the *P. sitchensis* map to the *P. abies* consensus map (Bernhardsson *et al*. 2019), corresponding to 1,935 *P. glauca* catalog genes and 1,873 *P. abies* genome scaffolds (Fig. 3a and b). Across marker pairs, 88% were assigned to the same linkage group with an average correlation in marker order of 0.96 (0.91–0.98). Synteny with the *P. flexilis* (limber pine) map (Liu *et al*. 2019) was based on 1,514 marker pairs with 1,397 unique *P. flexilis* markers on 1,397 unique *P. glauca* catalog genes with 85% of markers assigned to the same linkage group and an average correlation in marker order of 0.93 (0.88–0.97) (Fig. 3c). In all 3 comparisons, markers that did not align were distributed evenly across the 12 linkage groups (Fig. 3), and there were no indications of inversions or translocations in marker order within linkage groups (Supplementary Fig. 2).

### An integrated spruce map

**Fig. 4.**
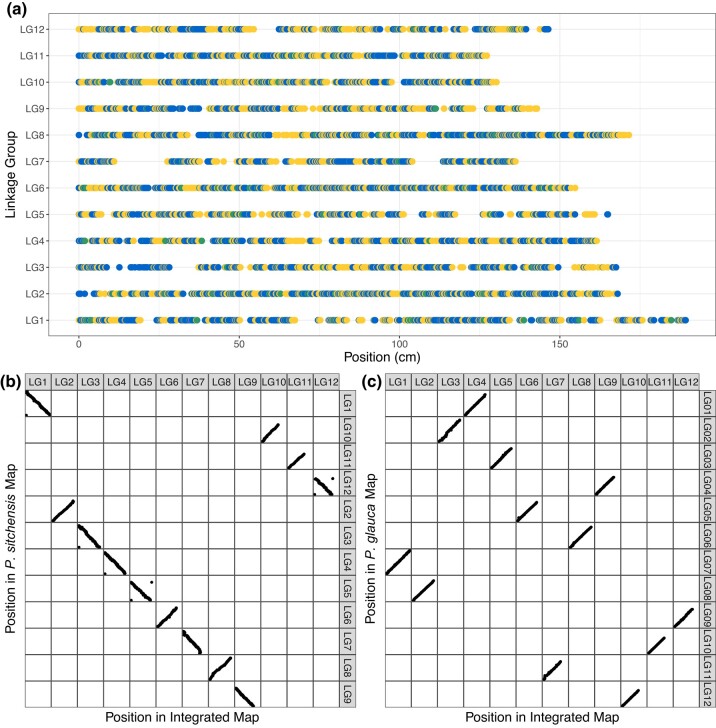
A representation of the integrated map of *P. sitchensis* and *P. glauca* and comparison to individual species maps. a) Chromosome or linkage group (LG) is on the *y*-axis with marker position on the *x*-axis. Points represent markers that are color coded for markers found in both species maps that were used to create an initial map of only overlapping markers (green) and markers found only in *P. sitchensis* (blue) or *P. glauca* (yellow) that were added in a second integration step to make the consensus integrated map. b, c) Comparison of marker assignment and order on linkage groups between the *P. sitchensis* (b) and *P. glauca* (c) maps (*x*-axis) to the integrated map (*y*-axis).

A total of 2,327 markers that were present in both the *P. glauca* and *P. sitchensis* maps were used to produce a merged map of overlapping markers. After selecting a single marker per gene in the reference *P. glauca* gene catalog from the *P. sitchensis* map and filtering for discrepancies between the *P. sitchensis* and *P. glauca* maps, 20,983 and 8,539 markers were selected to integrate the *P. sitchensis* and *P. glauca* maps, respectively, including 2,327 overlapping markers. After removing gaps at the terminal ends of linkage groups, the final integrated map contained 27,052 markers with 11,331 *P. glauca* catalog genes for a total map length of 1,860.2 cM and an average distance between markers of 0.07 cM. The integrated map placed an additional 6,195 *P. glauca* catalog genes compared to the *P. sitchensis* map and an additional 18,519 markers, including 2,798 additional *P. glauca* catalog genes, compared to the *P. glauca* map (Fig. 4a). All markers were assigned to the same linkage groups in both maps and concordance in marker order averaged 0.96 and 0.99 across linkage groups for the *P. sitchensis* and *P. glauca* maps, respectively (Fig. 4b and c).

## Discussion

The size and complexity of conifer genomes have limited the assembly of high-quality whole-genome sequences, as indicated by the high degree of fragmentation in early whole-genome assemblies (e.g. Birol *et al*. 2013; Nystedt *et al*. 2013; Zimin *et al*. 2014; Warren *et al*. 2015; De La Torre *et al*. 2019). Not surprisingly, conifer genome assemblies are still only available for species of high economic or ecological significance, and population-level genome resequencing is generally lacking. The importance and utility of linkage maps to assist assignments of large scaffolds to linkage groups were recently illustrated in a comparative genomic study focusing on *Picea* species, in conjunction with the use of long-read sequencing methods (Gagalova *et al*. 2022). Our *P. sitchensis* map is comparable in terms of the number of mapped genes (5,414) and superior based on the density of markers (21,570) to other recently expanded conifer genetic maps that integrated markers obtained by next-generation sequencing, such as those made for *P. glauca* (Gagalova *et al*. 2022; 14,727 expressed genes), *P. abies* (Bernhardsson *et al*. 2019; 21,506 markers containing 17,079 gene models), *P. flexilis* (Liu *et al*. 2019; 9,612 gene models), maritime pine (*P. pinaste*r) (Chancerel *et al*. 2013; ∼1,100 markers), and loblolly pine (*P. taeda*) (Westbrook *et al*. 2015; 3,856 markers across 3,305 genome scaffolds). Here, we discuss how our approach has simplified the development and integration of maps and highlight how the resulting resource can expand our understanding of conifer genomes and support genetic resource management.

### Map development and integration

In this study, by using 2 types of genotypic data and maps from 2 species, we were able to maximize the number of markers we mapped and gather and integrate a large amount of genomic information. This was made possible by combining RAD-seq and SNP array genotypic data for the same individuals of 2 unrelated Sitka spruce full-sib families. This allowed us to merge and order both marker types together, while also combining family data on the front end during map development, a feature unique to LepMap-3 (Rastas 2017). Earlier studies have relied on combining multiple marker types in forest trees but most often on a smaller scale. For example, AFLPs, ESTPs, SSRs, and gene-based SNPs were mapped together in *P. glauca* (831 markers and 348 genes) and in *P. mariana* (835 markers and 328 genes) (Pavy *et al*. 2008). Alternatively, several distinct maps produced with different marker types were reconstructed using gene-based SNPs, by using the same principles as applied here (Westbrook *et al*. 2015). In contrast, we obtained and used large SNP datasets from both exome sequencing and RAD-seq and by analyzing 2 independent full-sib families. This allowed us to produce individual component maps to verify for map coherence across genetic backgrounds before producing a consensus map with all data combined (Fig. 2). Recent high-density genetic maps have used a simpler approach based on a single marker type (Neves *et al*. 2014; de Miguel *et al*. 2015; Plomion *et al*. 2016; Pavy *et al*. 2017; Bernhardsson *et al*. 2019; Liu *et al*. 2019); however, our approach allowed us to draw inferences efficiently across marker types in a single step without requiring a map integration step.

By using an exome capture probe set designed and validated in *P. glauca* (Stival Senal *et al*. 2014) for SNP discovery in *P. sitchensis*, we explicitly aimed to obtain genotyping data in overlapping genomic sequences to enable direct comparisons across multiple conifer species. While this approach has been successfully used previously for SNP discovery across species, it had not yet been used to create an integrated map. The 4,590 SNP array markers and 1,094 RAD-seq markers were located in or matched a *P. glauca* transcriptome sequence, allowing us to compare our *P. sitchensis* consensus map robustly with the *P. glauca* map (Pavy *et al*. 2017). The gene-based markers also allowed comparison with linkage maps in *P. abies* (Bernhardsson *et al*. 2019) and *P. flexilis* (Liu *et al*. 2019). This comparison indicated the highest levels of synteny in *Picea*–*Picea* comparisons, with levels ranging as expected owing to respective pairwise phylogenetic distance, i.e. slightly lower synteny in the *Picea*–*Pinus* comparison. This is also the first study to integrate high-density linkage maps from 2 different conifer species, creating a resource that is more informative for each individual species. The integrated map provides information on conserved gene locations across species and provides a foundation for further development and integration with other species toward a more complete and comprehensive conifer genomic resource.

### Evolutionary insights and resource for breeding and conservation

We developed a high-density linkage map with 21,570 markers in *P. sitchensis* and an integrated map with 27,052 markers for *P. sitchensis* and *P. glauca*, both of which should facilitate further improvement of conifer genome sequence assemblies and contiguity. There is a high level of macro-synteny and macro-collinearity among species in the Pinaceae (e.g. Pavy *et al*. 2012; Westbrook *et al*. 2015). Here, we reported correlations of 0.93 or higher for the markers across species and genera. This apparent lack of chromosomal rearrangement enables genomic integration across species such as the creation of consensus genetic maps, as seen for *P. taeda* and *P. elliottii* with 3,856 shared markers (Westbrook *et al*. 2015). Many of the current conifer genome assemblies are still highly fragmented (e.g. Nystedt *et al*. 2013; Zimin *et al*. 2014; Gagalova *et al*. 2022) and contain many partial gene sequences (e.g. Warren *et al*. 2015), which leaves large gaps in our ability to conduct comparative genomic and evolutionary studies.

Recently, the integration of an expanded high-density linkage map from *P. glauca* and shotgun genome assemblies was reported in *P. glauca*, *Picea engelmannii*, *P. sitchensis*, and a natural hybrid of the 3 species (interior spruce) (Gagalova *et al*. 2022). Up to 32% of genome scaffolds could be anchored to linkage groups and further assembled into super-scaffolds representative of chromosomes, in addition to validating those areas of the genome assembly (Gagalova *et al*. 2022). Up to 65% of genomic scaffolds could be recently anchored on the *P. glauca* genetic map following improvement of genome assemblies using longer reads (R Warren and I Birol, University of British Columbia, personal communication). Therefore, the integrated linkage map generated herein will further improve this rate and inform genome assemblies more exhaustively, particularly for *P. sitchensis*, and facilitate cross-species comparisons among *Picea* spp. This will result in an improved structural characterization of conifer genomes including micro-rearrangements and the organization of genes in tandem arrays or functional operons (Pavy *et al*. 2017). This augmented spruce consensus linkage map has the potential to shed new insights on early lineage divergence and their genomic footprints in the conifers, such as between the Pinaceae and Cupressaceae (Moriguchi *et al*. 2012, de Miguel *et al*. 2015) and Taxaceae families, with the recent release of genome assemblies for *Sequoiadendron giganteum* (Lindl.) J. Buchh (Scott *et al*. 2020), *Sequoia sempervirens* Endl. (Neale *et al*. 2022), and *Taxus chinensis* (Pilger) Rehd. (Xiong *et al*. 2021).

We have discovered 286,159 SNPs distributed across 23,480 *P. sitchensis* gene clusters after exome capture and sequencing, with probes developed on *P. glauca* (Stival Sena *et al*. 2014), and previously validated in *P. mariana* (Pavy *et al*. 2016) and *P. abies* (Azaiez *et al*. 2018). The highly conserved nature of gene coding sequences across these congeneric species made it possible to successfully transfer probes between taxa and suggests that the set of probes used in this study should work across the whole *Picea* genus. By targeting common genes across species, our SNP discovery approach allowed us to develop a genotyping array that selectively included genes that were both unmapped and previously mapped in *P. glauca* (Pavy *et al*. 2017). This approach aimed to facilitate direct cross-species comparisons by using previously mapped genes as anchoring points and filling gaps in previous maps by positioning unmapped genes. This strategy has resulted in an integrated map including 27,052 markers, which will offer an opportunity to increase our understanding of gene family evolution in conifers and plants more broadly. For example, in conifers, stress-related gene families have been reportedly more diverse than in Angiosperms (Rigault *et al*. 2011; Warren *et al*. 2015; De la Torre *et al*. 2019; Gagalova *et al*. 2022), and a high level of sequence novelty was found across conifer species in dehydrin (Stival Sena et al. 2018), leucine-rich repeat (NLR) (Van Ghelder *et al*. 2019), and R2R3-MYB (Bedon *et al*. 2010) gene families, among others. In the conifer *P. flexilis*, linkage mapping showed that disease resistance NLR genes were highly clustered on a few linkage groups (Liu *et al*. 2019). Therefore, new linkage maps and integrated genomic resources (as reported here or in Gagalova *et al*. 2022) should help to further our understanding of the evolution of this large gene family.

From an applied perspective, the resources developed herein will also support genetic resource management in *P. sitchensis*, the dominant productive forestry species in the British Isles. These resources include a genotyping array of 12,911 SNPs, of which 5,414 were polymorphic (Supplementary Data 2) and produced large-scale genotypic data for 2 mapping families and a breeding population (Fig. 1), as well as high-density linkage maps. The SNPs were discovered in gene coding regions, which are conserved across species and contain few repetitive sequences compared to intergenic regions; therefore, the polymorphic SNPs may be amplified using other PCR techniques with a predicted high success rate. Genomic selection is poised to accelerate and transform forest tree breeding, although its implementation in breeding programs targeting both gymnosperm and angiosperm trees remains challenging (Grattapaglia 2022). In conifers such as *Pinus* spp. and *Picea* spp., genomic selection gave genetic prediction abilities approaching those achieved with pedigree-based selection for growth, wood properties, and insect resistance (Beaulieu *et al*. 2014; Lenz *et al*. 2020; Bousquet *et al*. 2021; Calleja-Rodriguez *et al*. 2020; Isik 2022 ). However, the high cost associated with the acquisition of large-scale genotypic and phenotypic data still represents a barrier for the routine use of genomic prediction in tree breeding programs (Klápště *et al*. 2022). In a companion study to this report, Ilska *et al*. (2023) have used the newly developed *P. sitchensis* linkage map to impute missing RAD-seq genotypes in mapping families 1 and 2 and in 1 unrelated family, which resulted in improved call rates by up to 10%, and a significant reduction of genotyping costs by allowing the use of lower-cost genotyping methods, which performance in genomic selection schemes is generally negatively affected by lower call rates and genome coverage. The genotypes obtained in the current *P. sitchensis* breeding population have been analyzed in distinct genomic selection studies of growth and wood traits (Ilska *et al*. 2023) and for developing a low-cost DNA-fingerprinting assay (Godbout *et al*. 2017).

The linkage maps presented here will also facilitate mapping quantitative trait loci for traits related to adaptation (Pelgas *et al*. 2011; Pavy *et al*. 2017; Laoué *et al*. 2021) and pest resistance (Lind *et al*. 2014), among others, and comparative studies of genomic architecture to better understand the divergent or convergent nature of the evolution across spruces and other conifers. It would also allow to identify new candidate genes for further investigations at the functional level or for diagnostic marker development. Finally, evolutionary studies using linkage maps to gain insights into the structure of large gene families involved in disease resistance such as nucleotide-binding and NLR genes (Liu *et al*. 2019; Van Ghelder *et al*. 2019) or dehydrin genes for drought response (Stival Sena *et al*. 2018) may result in potent diagnostic tools and benefit forest practitioners involved in the management and the conservation of genetic resources in natural and breeding populations, especially in the context of accelerated climate change.

### Conclusion

In this study, we developed a highly densified genetic linkage map in *P. sitchensis* by efficiently combining different marker types and by targeting gene coding regions to facilitate comparative genomic analyses and integration across species (Fig. 2). Together, the newly identified SNP markers and new genetic linkage maps will help improving genome assemblies, expanding our understanding of conifer genome evolution, and supporting *P. sitchensis* genomic resource development and genetic resource management. Gymnosperms have been reported to have less diverse and less dynamic genomes compared to those of flowering plants (Leitch and Leitch 2012), but interestingly, they are genetically diverse and have a large proportion of their rapidly evolving genes related to stimuli and stress response (Gagalova *et al*. 2022), many of which belong to highly diversified gene families (e.g. Bedon *et al*. 2010; Stival Sena *et al*. 2018; Van Ghelder *et al*. 2019). Conifers are also characterized by high levels of intraspecific phenotypic variability in defensive compounds (e.g. Mageroy *et al*. 2015; Parent *et al*. 2020; Tumas *et al*. 2021). The resources reported here will aid the understanding, conservation, and sustainable use of this wealth of adaptive potential to support the resilience of natural and breeding populations in the face of climate change.

## Supplementary Material

jkae020_Supplementary_Data

## Data Availability

Genotype tables for each map (i.e. RAD, Chip, and RAD-Chip maps), pedigree files, code to convert files to LepMap3 format, final map files for the 3 maps and the integrated map, and SNP array information are available at Dryad (https://doi.org/10.5061/dryad.ghx3ffbv6). Codes described to develop linkage maps, compare composite maps and species maps, and develop the integrated map are publicly available at https://github.com/HayleyTumas/SitkaLinkageMap. Supplemental material available at G3 online.
