# Supplementary material for: High-density genetic linkage mapping in Sitka spruce advances the integration of genomic resources in conifers
Source: G3 (Bethesda). 2024 Feb 15;14(4):jkae020. doi: 10.1093/g3journal/jkae020 (PMC10989875; doi:10.1093/g3journal/jkae020)
Supplement: jkae020_Supplementary_Data [file jkae020_supplementary_data.zip › Supplemental_Material_G3-2023-404751.pdf]

High-density genetic linkage mapping in Sitka spruce advances the integration of genomic resources in conifers

Hayley Tumas[1*], Joana J. Ilska[2], Sebastien Gérardi[3,4], Jerome Laroche[4], Stuart A'Hara[5], Brian Boyle[4], Mateja Janes[2], Paul McLean[5], Gustavo Lopez[5], Steve J. Lee[5], Joan Cottrell[5], Gregor Gorjanc[2], Jean Bousquet[3,4], John A. Woolliams[2], John J. MacKay[1*]

[1]Department of Department of Biology, University of Oxford, South Parks Road, Oxford, OX1 3RB, UK.

[2]The Roslin Institute, Royal (Dick) School of Veterinary Science, University of Edinburgh, Easter Bush, Midlothian, EH25 9RG, Scotland, UK.

[3]Canada Research Chair in Forest Genomics, Forest Research Centre, Université Laval, Québec, QC, GIV 0A6, Canada

[4]Institute for Systems and Integrative Biology, Université Laval, Québec, QC, GIV 0A6, Canada

[5]Forest Research, Northern Research Station, Roslin, Midlothian, EH25 9SY, UK.

Data S1 – SNP calling, marker parameters, and map comparisons.

1. Platypus, default parameters used in this study for SNP calling.

Platypus Options ={'assemblyRegionSize': 1500, 'trimReadFlank': 0, 'assembleBadReads': 1, 'bamFiles': ['List_bam'], 'minVarDist': 9, 'trimSoftClipped': 1, 'minReads': 25, 'qualBinSize': 1, 'refFile': '/home/jelar5/consultations/spruce-up/platypus/GCAT/refgenome/GCAT_WS-3.3.cluseq', 'maxHaplotypes': 50, 'filterVarsByCoverage': 0, 'maxSize': 1500, 'originalMaxHaplotypes': 50, 'skipDifficultWindows': 1, 'parseNCBI': 0, 'skipRegionsFile': None, 'noCycles': 0, 'trimAdapter': 0, 'minPosterior': 5, 'assembleAll': 1, 'trimOverlapping': 1, 'filterDuplicates': 0, 'abThreshold': 0.001, 'minFlank': 10, 'bufferSize': 100000, 'fileCaching': 0, 'useEMLikelihoods': 0, 'coverageSamplingLevel': 30, 'calculateFlankScore': 0, 'logFileName': 'platypus_log.txt', 'nCPU': 8, 'filterReadsWithUnmappedMates': 0, 'qdThreshold': 10, 'maxVariants': 2, 'scThreshold': 0.95, 'filterReadsWithDistantMates': 0, 'maxReads': 5000000, 'badReadsWindow': 11, 'genIndels': 1, 'largeWindows': 0, 'minMapQual': 20, 'maxVarDist': 15, 'maxGOF': 20, 'rlen': 500, 'minGoodQualBases': 20, 'refCallBlockSize': 1000, 'countOnlyExactIndelMatches': 0, 'longHaps': 0, 'HLATyping': 0, 'filterReadPairsWithSmallInserts': 0, 'minBaseQual': 20, 'getVariantsFromBAMs': 1, 'genSNPs': 1, 'assemble': 0, 'assemblerKmerSize': 15, 'minVarFreq': 0.05, 'alignScoreFile': '', 'verbosity': 2, 'sourceFile': None, 'compressReads': 0, 'rmsmqThreshold': 20, 'filteredReadsFrac': 0.7, 'outputRefCalls': 0, 'badReadsThreshold': 10, 'hapScoreThreshold': 15, 'regions': None, 'sbThreshold': 0.01, 'output': 'SpruceUP_GCAT_Pcarto_PopBreeding.vcf', 'assembleBrokenPairs': 0, 'mergeClusteredVariants': 1, 'maxGenotypes': 1275, 'nInd': 3}

2. *Pices sitchensis* marker analysis parameters and map comparison details

Table S1. The lodLimit that assigned the most markers to chromosomes and was used for the SeparateChromosomes2 and JoinSingles2All steps of LepMap3 v0.2 for each map. Note that family maps were made separately for the RAD-Chip map and that the composite map of both families was completed a second time after identifying and removing consistently mismatching markers (see Methods and the GitHub repository).

Map	SeparateChromosomes2	JoinSingles2All
RAD Map	20	10
Chip Map	41	11
RAD-Chip Map Family 1	45	10
RAD-Chip Map Family 2	30	8
RAD-Chip Map Composite	30	10
RAD-Chip Map Composite without mismatch markers	28	10

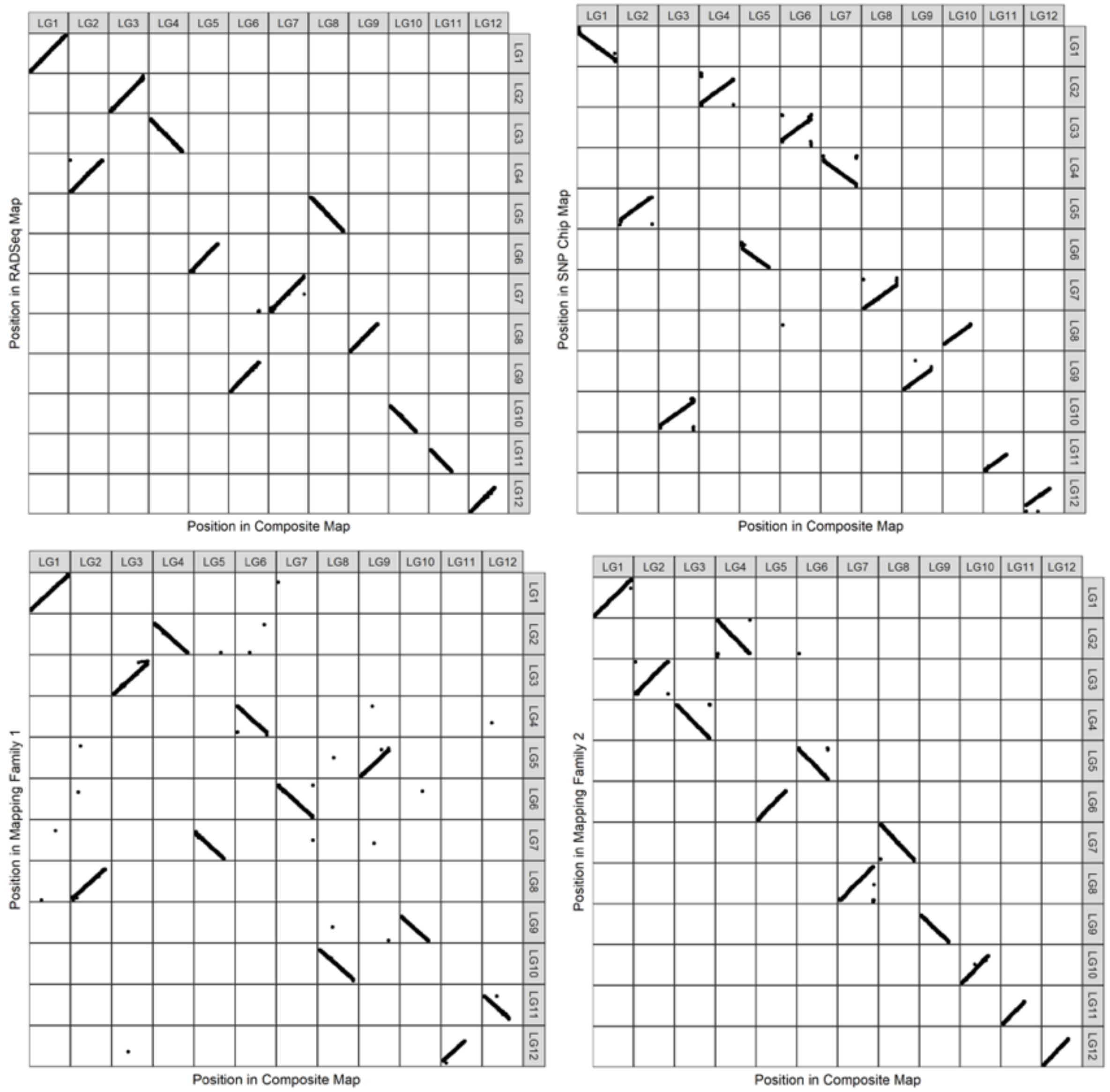

Figure S1. Comparison of marker assignment and order in chromosomes (linkage groups) in *P. sitchensis* between the consensus (Composite) RAD-Chip map (x-axis) and the four component maps (y-axis) made using data from: both full-sib families and only RAD-Seq markers (RAD map, top left); both families and only SNP Chip markers (SNP Chip map, top right); only samples from family 1 and the combined marker dataset (Fam1, bottom left); only samples from family 2 and the combined marker datatset (Fam2, bottom right). Each grid cell represents a linkage group (LG) with markers represented as points. Markers that group on the same chromosome in similar order fall along a linear diagonal in the same grid cell, while markers that group on different linkage groups appear as singular points in other grid cells. Note that corresponding chromosomes may be assigned different linkage group numbers across maps, i.e., LG1 in one map may not be the same chromosome as LG1 in a different map.

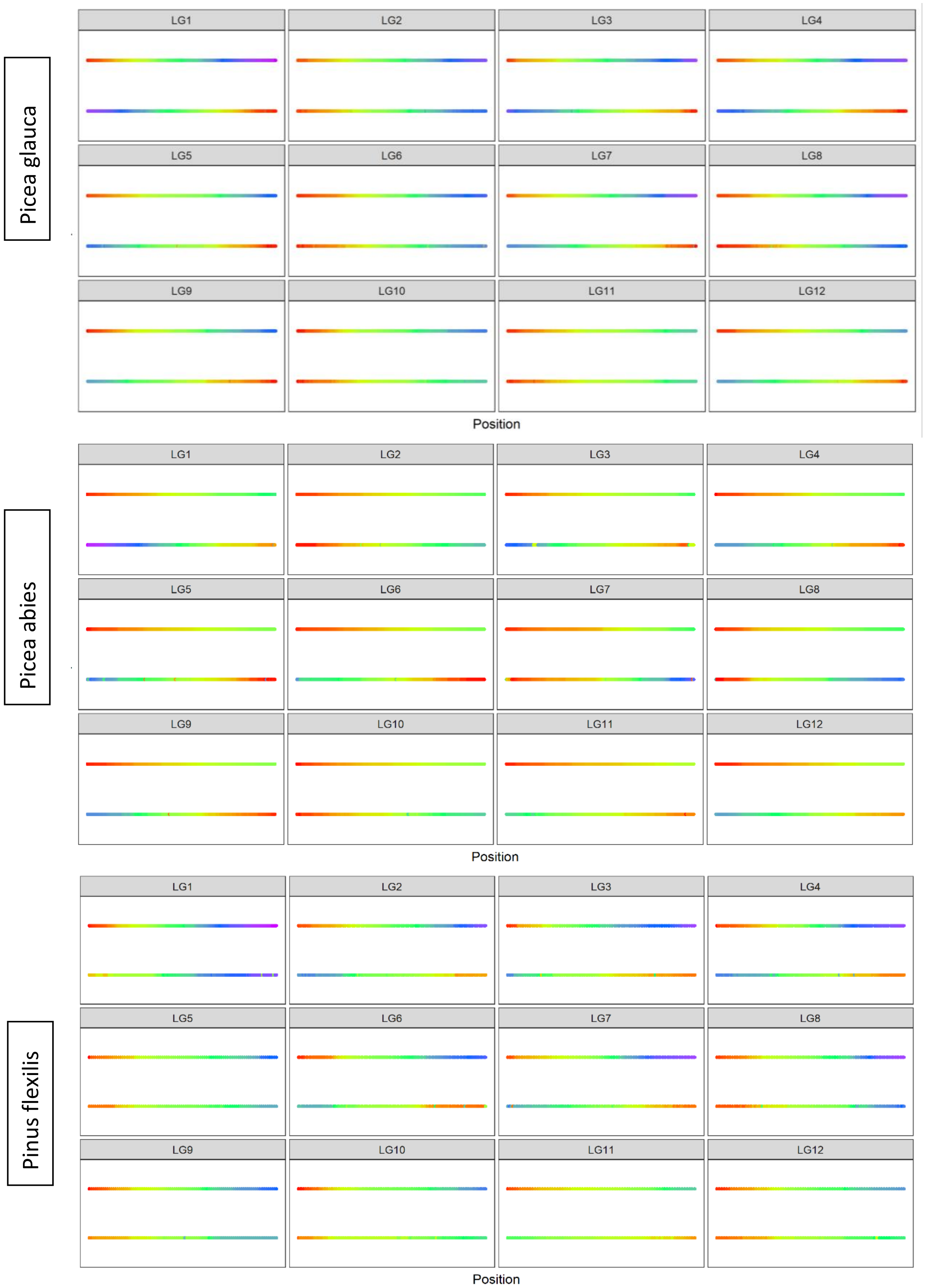
Picea glauca
LG1
LG2
LG3
LG4
LG5
LG6
LG7
LG8
LG9
LG10
LG11
LG12
Position
Picea abies
LG1
LG2
LG3
LG4
LG5
LG6
LG7
LG8
LG9
LG10
LG11
LG12
Position
Pinus flexilis
LG1
LG2
LG3
LG4
LG5
LG6
LG7
LG8
LG9
LG10
LG11
LG12
Position

Figure S2. Graphs used to visually check for inversions when comparing markers mapped in *P. sitchensis* to other Pinaceae as indicated. Markers were assigned a color from red to blue based on the order in their species map then graphed in the order they were mapped in *P. sitchensis* (top line in each plot). A larger inversion would be indicated by a section of the species line (bottom line in each plot) showing colors in opposite order (i.e. if the species line goes from red to blue an inversion would be a section that has blue to red colors). Although some markers are in a different order, no inversions were seen.
